# Increased placental soluble fms-like tyrosine kinase receptor-1 (sFLT1) drives the antiangiogenic profile of maternal serum preceding preeclampsia but not fetal growth restriction

**DOI:** 10.1161/HYPERTENSIONAHA.122.19482

**Published:** 2022-07-22

**Authors:** Francesca Gaccioli, Ulla Sovio, Sungsam Gong, Emma Cook, D Stephen Charnock-Jones, Gordon CS Smith

**Affiliations:** 1 Department Obstetrics and Gynaecology, University of Cambridge, Cambridge, UK; 2 Centre for Trophoblast Research, University of Cambridge, Cambridge, UK

**Keywords:** preeclampsia, fetal growth restriction, placenta growth factor, sFLT1 protein, angiogenesis, cohort studies, placenta

## Abstract

**Background:**

Preeclampsia and fetal growth restriction (FGR) are both associated with an increased ratio of soluble fms-like tyrosine kinase-1 (sFLT1) to placenta growth factor (PlGF) in maternal serum. In preeclampsia, it is assumed that increased placental release of sFLT1 results in PlGF being bound and inactivated. However, direct evidence for this model is incomplete and it is unclear if the same applies in FGR.

**Methods:**

We conducted a prospective cohort study where we followed 4,212 women having first pregnancies from their dating ultrasound, obtained blood samples serially through the pregnancy and performed systematic sampling of the placenta after delivery. The aim of the present study was to determine the relationship between protein levels of sFLT1 and PlGF in maternal serum measured at ~36 weeks and placental tissue lysates obtained after term delivery in 82 women with preeclampsia, 50 women with FGR and 132 controls.

**Results:**

The sFLT1:PlGF ratio was increased in both preeclampsia and FGR in both the placenta and maternal serum. However, in preeclampsia the maternal serum ratio of sFLT1:PlGF was strongly associated with placental sFLT1 level (r=0.45, P<0.0001) but not placental PlGF level (r=-0.17, P=0.16). In contrast, in FGR the maternal serum ratio of sFLT1:PlGF was strongly associated with placental PlGF level (r=-0.35, P=0.02) but not sFLT1 level (r=0.04, P=0.81).

**Conclusions:**

We conclude that the elevated sFLT1:PlGF ratio is primarily driven by increased placental sFLT1 in preeclampsia whereas in FGR it is primarily driven by decreased placental PlGF.

## Acronyms

FGR: fetal growth restriction
PE: preeclampsia
CON: control
wkGA: weeks of gestational age
RNA-seq: RNA sequencing
FLT1: fms-like tyrosine kinase
sFLT1: soluble FLT1
PlGF: placenta growth factor
VEGFR: vascular endothelial growth factor receptor
POP study: Pregnancy Outcome Prediction study
ACOG: American College of Obstetricians and Gynecologists
HELLP: Hemolysis/Elevated Liver enzymes/Low Platelet
MoM: multiple of the median
FTE: full time education
AC: abdominal circumference
PI: pulsatility index

## Introduction

Preeclampsia is an acquired disorder of pregnancy, manifested by hypertension combined with dysfunction of one or more other organs, typically including the kidney. The clinical presentation of preeclampsia is preceded by elevated circulating soluble fms-like tyrosine kinase-1 (sFLT1) and decreased placenta growth factor (PlGF), a pro-angiogenic and pro-endothelial growth factor which is bound and inactivated by sFLT1. These associations support the clinical use of the maternal serum sFLT1:PlGF ratio (or PlGF on its own) to rule in or rule out preeclampsia in women presenting with features suggestive of the disease^1^ and this has been included in clinical guidelines in the UK and other European countries.^2^

Although complete mechanistic insight into the pathophysiology of preeclampsia remains elusive, one current model is that the disease is causally associated with elevated sFLT1 in the maternal circulation.^3^ It is hypothesized that increased placental release of sFLT1 results in elevated maternal serum levels of the protein, which then binds and inactivates PlGF in the maternal circulation, and it may also act directly on endothelial cells. Low maternal PlGF and/or reduced vascular endothelial growth factor receptor (VEGFR) signalling results in endothelial dysfunction, a cardinal element of the multi-system manifestations of the condition. We and others have also shown that both the sFLT1:PlGF ratio and PlGF on its own are also predictive of fetal growth restriction (FGR), defined as failure of a fetus to achieve its genetically determined growth potential.^4, 5^ However, studies have reported increased PlGF mRNA in the FGR placenta,^6^ unchanged levels,^7^ or reduced expression.^8^ Moreover, sFLT1 mRNA and protein expression has been shown to be increased in FGR.^9^ Furthermore, in both preeclampsia and FGR, sFLT1 and PlGF may originate from non-placental sources and altered maternal serum levels of sFLT1 and PlGF may be unrelated to the placenta.^10–15^ Hence, the relationship between placental expression of angiogenic regulators and low maternal serum levels of PlGF and elevated sFLT1:PlGF ratio in PE and FGR is currently unclear.

The aim of the present study was to determine the relationship between placental sFLT1 and PlGF (assessed following birth) and maternal serum levels of sFLT1 and PlGF in late pregnancy in women giving birth at term with or without a diagnosis of preeclampsia or fetal growth restriction. We used a large prospective cohort of nulliparous women who had serial blood sampling throughout pregnancy, deep phenotyping of pregnancy outcome, and systematic sampling of the placenta following delivery.

## Materials and Methods

The data that support the findings of this study are available from the corresponding author upon reasonable request.

### Study design

The Pregnancy Outcome Prediction (POP) was a prospective cohort study of 4,212 nulliparous women attending the Rosie Hospital (Cambridge, United Kingdom) for their dating ultrasound scan between January 14, 2008, and July 31, 2012 inclusive, with a viable singleton pregnancy.^16–18^ Briefly, pregnant women had blood taken at the booking visit at ˜12 weeks of gestation (wkGA) and at 3 subsequent visits (at ˜20wkGA, ˜28wkGA, and ˜36wkGA), when ultrasound scans were also performed. Placentas were collected after delivery. All participants provided written informed consent and ethical approval was given by the Cambridgeshire 2 Research Ethics Committee (reference number 07/H0308/163).

A total of 3,890 women in the study had placental biopsies obtained following delivery, and 1,476 (38%) had the placental biopsy obtained within 30 minutes of the delivery. Among these, we studied a cohort of women which was previously described and included 82 preeclampsia cases (defined on the basis of the 2013 ACOG criteria)^19^ and 82 matched healthy term pregnancies.^20^ Healthy pregnancies had a live born infant with a birth weight percentile in the normal range (20-80^th^ percentile) and no evidence of slowing fetal growth trajectories, hypertension, preeclampsia, Hemolysis/Elevated Liver enzymes/Low Platelet (HELLP) syndrome, gestational diabetes or diabetes mellitus type I or type II or other obstetric complications. Case-control matching was performed as closely as possible on the following: presence of labor, gestational age, fetal sex, caesarean section, smoking status, maternal BMI and maternal age. We studied a second cohort of women who delivered at term, including healthy pregnancies with appropriate for gestational age fetuses (defined as above) and cases with fetal growth restriction (FGR, defined as a birth weight <10^th^ centile and ≥1 of the following: top decile of uterine Doppler pulsatility index (PI), top decile of umbilical Doppler PI and bottom decile of abdominal circumference growth velocity, as previously described)^21^ (n=50/group). Nine patients were included in both cohorts: 6 with healthy pregnancies and 3 with both preeclampsia and a FGR fetus. The median collection time (min to max) for placental samples was 40.3 weeks (37.0 weeks to 42.3 weeks).

### Enzyme-linked immunosorbent assay (ELISA) of placental proteins

Placental tissues (approximately 5-10mg/biopsy) were taken from the maternal surface of the placenta following removal of decidual contamination and stored at -80°C for subsequent analysis. Samples were lysed in Lysing Matrix D tubes (MP Biomedicals) and the protein concentration measured with the BCA Protein Assay Kit (ThermoFisher Scientific). The lysates were diluted to a protein concentration of 1μg/μl and placental sFLT1 and PlGF quantified using enzyme-linked immunosorbent assays (R&D Systems, cat DVR100B and DPG00, respectively), following the manufacturer's protocols. It should be noted that the sFLT1 ELISA measures both soluble and membrane bound forms of FLT1 in tissue samples. However, in the placenta sFLT1 dominates over the membrane anchored FLT1^22^ and this is consistent with our results demonstrating that the sFLT1 mRNA variant accounts for ˜90% of the placental FLT1 transcripts (Supplemental Results). Therefore for simplicity we describe all these measurements as the sFLT1 levels. The PlGF ELISA measures free PlGF.^3, 23^

### Maternal serum immunoassays

Circulating levels of sFLT1 and PlGF were measured in maternal serum samples at 36wkGA (median collection time (min to max): 36.1 weeks (35.0 to 37.6 weeks)) using Roche Elecsys assays on the electro-chemiluminescence immunoassay platform Cobas e411 (Roche Diagnostics) as previously described.^24^ With this system, the intra-assay coefficient of variation for human serum samples is <2% for both the assays, and the inter-assay coefficients of variation are 2.3-4.3% and 2.7-4.1% for the sFLT1 and PlGF assay, respectively. The Elecsys PlGF assay measures biologically active PlGF in serum samples.^25^ The Elecsys sFLT1 assay detects total sFLT1 and is unaffected by PlGF binding.^25^ Assays were performed blind to the patients’ clinical information and pregnancy outcomes. The associations between measurements of the proteins in maternal serum in the whole cohort have previously been described in relation to both preeclampsia and FGR^4, 24^ and here we report the values for the sub-groups in which we measured placental protein levels.

### Statistical analysis

Data management and statistical analyses were performed using Stata v17 (StataCorp LLC) and GraphPad Prism version 9.2.0 (GraphPad Software LLC). Placental protein levels were expressed as Z scores of the log-transformed concentrations. Maternal serum proteins were expressed as the multiple of the median (MoM) of control samples (adjusted for gestational age, maternal weight and storage time at measurement), log-transformed and turned into Z scores, referent to the whole POP study cohort. The unadjusted sFLT1:PlGF ratio was log-transformed and turned into a Z score. P values were obtained using paired or unpaired 2-tailed t-test. Relationships between placental and maternal protein levels were determined using Pearson’s correlation coefficients.

## Results

From the first cohort of 82 cases of preeclampsia and 82 healthy pregnancies, (i) 2 pairs had placental sFLT1 levels below the assay detection limit, and (ii) 14 pairs had missing maternal values. Therefore, in this cohort we studied placental sFLT1 and PlGF, and maternal protein levels in 160, 164 and 136 patients, respectively. From our second cohort of 100 pregnancies with appropriate for gestational age and FGR fetuses, (i) 3 patients had placental sFLT1 levels below the assay detection limit, and (ii) 6 had missing maternal values. Therefore, in this cohort we studied placental sFLT1 and PlGF, and maternal protein levels in 97, 100 and 94 patients, respectively. Clinical characteristics are presented in Table 1.

**Figure 1:**
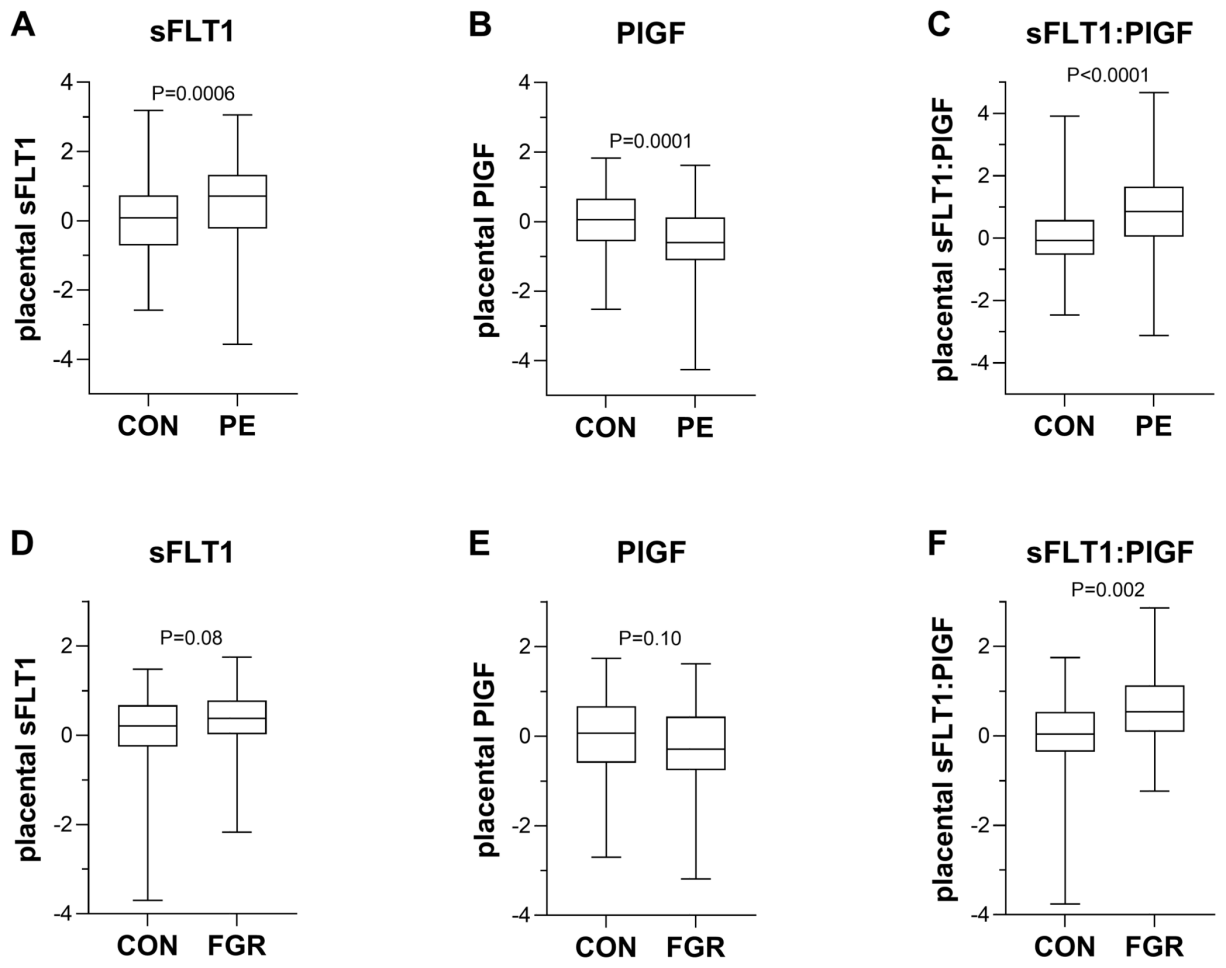
Placental protein levels of sFLT1, PlGF and the Flt1:PlGF ratio in pathological and healthy pregnancies. Protein levels were measured in term placentas from healthy pregnancies compared to (A-C) paired preeclamptic pregnancies (n=160 for sFLT1 and sFLT1:PlGF; n=164 for PlGF); (D-F) pregnancies with FGR fetuses (n=97 for sFLT1 and sFLT1:PlGF; n=100 for PlGF). Samples were omitted from the analyses if measurements were not available or below the detection limit of the assay and protein levels are expressed as Z scores of the log-transformed concentrations. Boxes indicate the median, 25th and 75th percentiles. Whiskers extend to the minimum and maximum values. P values, obtained using paired (A-C) or unpaired (D-F) 2-tailed t-test, are reported. sFLT1: soluble fms-like tyrosine kinase 1; PlGF: placental growth factor; CON: control/healthy pregnancy; PE: preeclampsia; FGR: fetal growth restriction.

**Figure 2:**
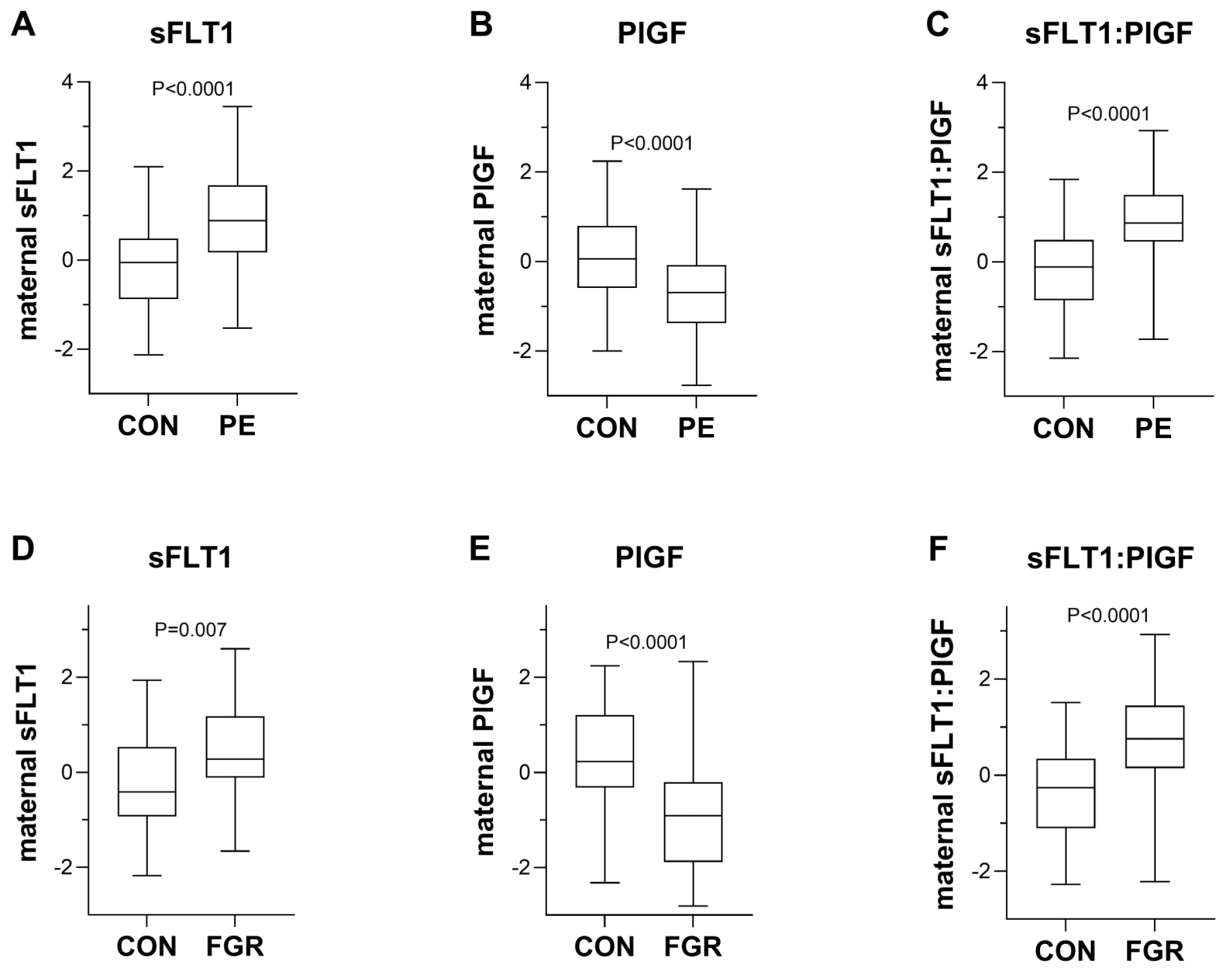
Maternal circulating levels of sFLT1, PlGF and the sFlt1:PlGF ratio in pathological and healthy pregnancies. Maternal serum protein levels were measured at ~36 weeks of gestation in two cohorts: (A-C) 68 paired healthy and preeclamptic pregnancies (n=136); (D-F) control pregnancies (n=48) and with FGR fetuses (n=46). Samples were omitted from the analyses if measurements were not available or below the detection limit of the assay. Maternal proteins are expressed as the multiple of the median (MoM) of control samples (adjusted for gestational age, maternal weight and storage time at measurement), log-transformed and turned into Z scores. The unadjusted sFLT1:PlGF ratio was log-transformed and turned into a Z score. Boxes indicate the median, 25th and 75th percentiles. Whiskers extend to the minimum and maximum values. P values, obtained using paired (A-C) or unpaired (D-F) 2-tailed t-test, are reported. sFLT1: soluble fms-like tyrosine kinase 1; PlGF: placental growth factor; CON: control/healthy pregnancy; PE: preeclampsia; FGR: fetal growth restriction.

Comparing placental protein levels in cases and controls, preeclampsia was associated with increased levels of sFLT1 protein, decreased levels of PlGF and an increased placental sFLT1:PlGF ratio (Figure 1A-C). Fetal growth restriction was not associated with altered sFLT1 and PlGF protein levels in the placenta, but sFLT1:PlGF was elevated in FGR placentas compared with controls (Figure 1D-F). Comparing maternal serum proteins at ˜36wkGA, both preeclampsia and FGR were associated with increased levels of sFLT1 protein, decreased levels of PlGF and an increased placental sFLT1:PlGF ratio (Figure 2).

We explored the effect of labor on the placental protein expression of PlGF and sFLT1 by comparing patients with >6h labor and those with shorter labor. Although we observed lower placental PlGF levels with prolonged labor, this is unlikely to explain our observations as there were similar proportions of cases and controls having labor <6h or >6 hours (Supplemental Results and Figure S1).

**Figure 3:**
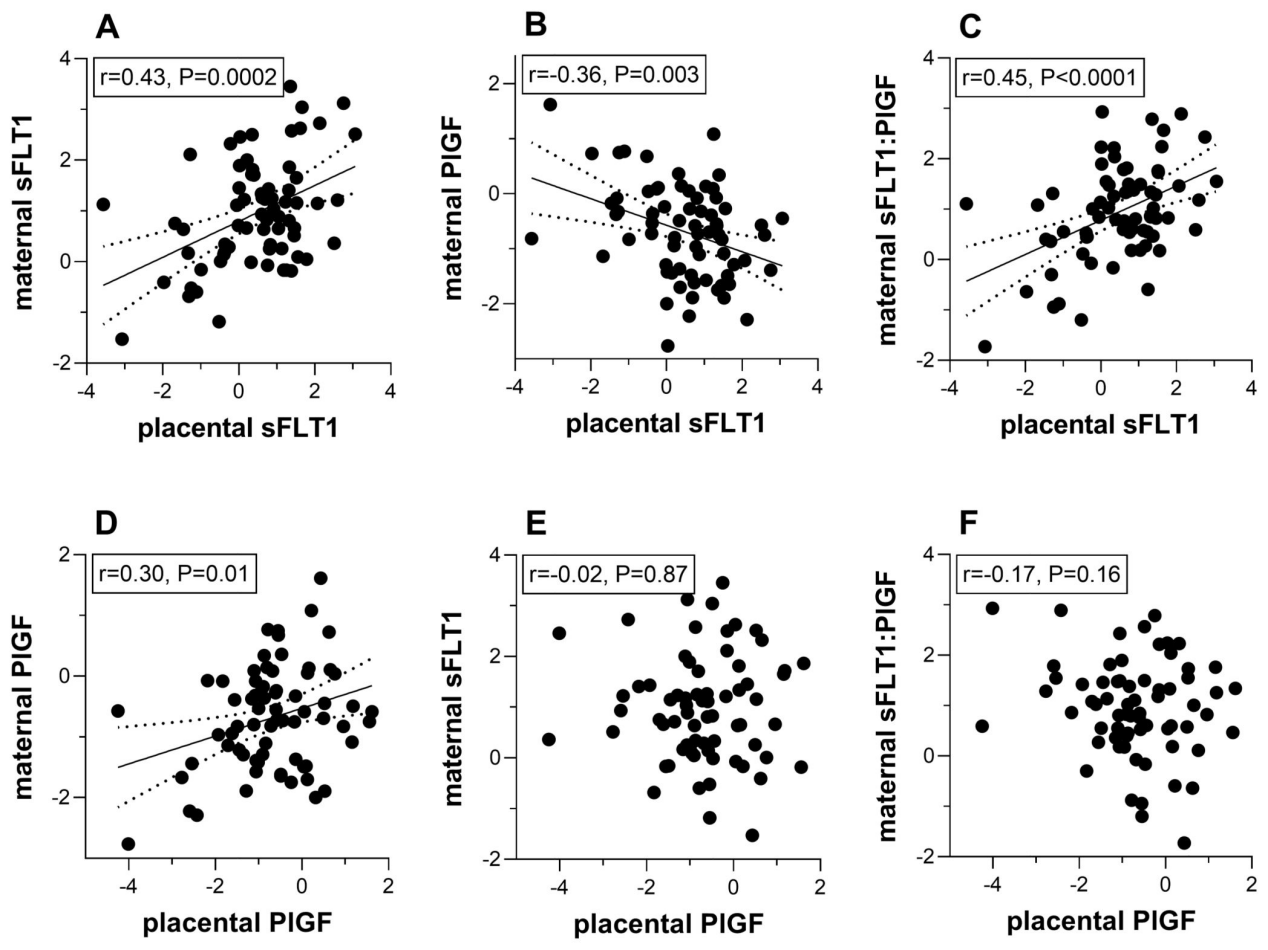
Relationship between placental sFLT1 and PlGF concentrations and maternal serum levels of sFLT1, PlGF and the sFLT1:PlGF ratio in pregnancies with preeclampsia. Maternal sFLT1, PlGF and the sFLT1:PlGF ratio at 36 weeks of gestation are plotted against term placental sFLT1 (A-C) or PlGF concentrations (D-F) in 69 women who ultimately delivered with a diagnosis of preeclampsia. Samples were removed from the analyses if measurements were not available or below the detection limit of the assay. Maternal proteins are expressed as the multiple of the median (MoM) of control samples (adjusted for gestational age, maternal weight and storage time at measurement). Then MoM values and placental protein concentrations were log transformed and expressed as Z scores. Best fitting regression line (solid) and 95% confidence bands (dotted) are indicated. Text boxes report Pearson’s correlation coefficients (r) and P values. sFLT1: soluble fms-like tyrosine kinase 1; PlGF: placental growth.

We next examined the correlation between placental protein levels measured from samples obtained immediately after birth and the maternal serum levels of the same proteins measured prior to birth at ˜36wkGA. It should be noted that RNA-Seq analysis of 169 of the placental samples included in this study demonstrated that tissue and maternal protein concentrations strongly correlated with their corresponding placental mRNA levels (Supplemental Methods and Figure S2). Among controls, there were no statistically significant relationships between placental sFLT1 or PlGF and maternal serum sFLT1, PlGF, or the sFLT1:PlGF ratio measured at ˜36wkGA (all p>0.05, data not shown). In contrast, among women who had a diagnosis of preeclampsia, there were strong correlations between the placental concentrations of sFLT1 and circulating sFLT1, PlGF, and the sFLT1:PlGF ratio at ˜36wkGA (Figure 3). However, placental PlGF was only associated with maternal serum PlGF and there was no correlation with maternal serum levels of sFLT1 or the sFLT1:PlGF ratio. Interestingly, among women with preeclampsia, placental sFLT1 concentration was a stronger predictor of maternal serum PlGF levels than placental PlGF expression.

**Figure 4:**
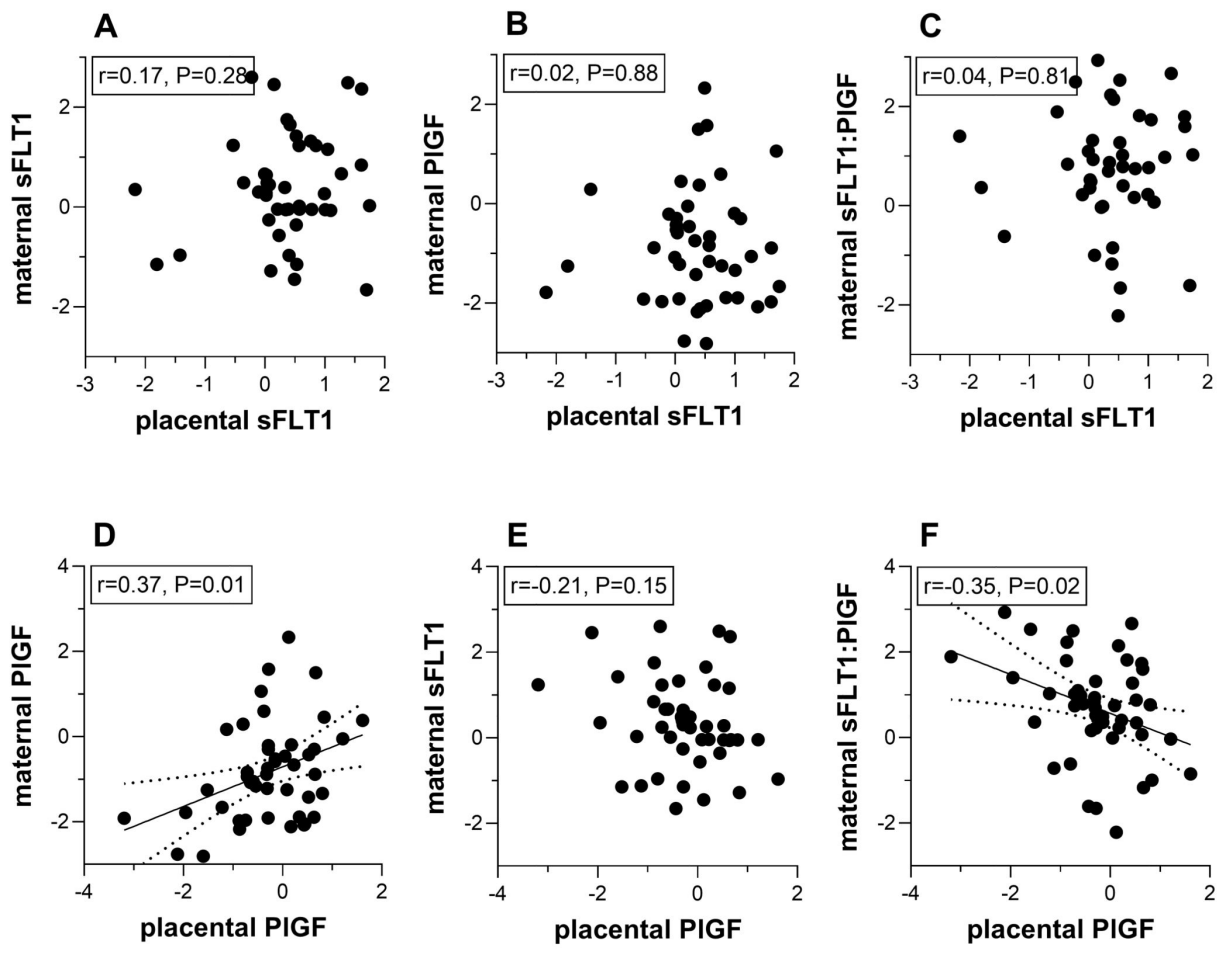
Relationship between placental sFLT1 and PlGF concentrations and maternal serum levels of sFLT1, PlGF and the sFLT1:PlGF ratio in pregnancies with fetal growth restriction. Maternal sFLT1, PlGF and the sFLT1:PlGF ratio at 36 weeks of gestation are plotted against term placental sFLT1 (n=44, A-C) or PlGF concentrations (n=46, D-F) in pregnancies with fetal growth restriction. Samples were removed from the analyses if measurements were not available or below the detection limit of the assay. Maternal proteins are expressed as the multiple of the median (MoM) of control samples (adjusted for gestational age, maternal weight and storage time at measurement). Then MoM values and placental protein concentrations were log transformed and expressed as Z scores. Best fitting regression line (solid) and 95% confidence bands (dotted) are indicated. Text boxes report Pearson’s correlation coefficients (r) and P values. sFLT1: soluble fms-like tyrosine kinase 1; PlGF: placental growth.

In pregnancies complicated by FGR, there was no association between placental sFLT1 level and maternal circulating sFLT1, PlGF, and the sFLT1:PlGF ratio at ˜36wkGA (Figure 4A-F). However, there was a strong positive correlation between placental PlGF and maternal serum PlGF and a strong inverse correlation between placental PlGF and maternal serum sFLT1:PlGF.

The observed correlations were detected despite the fact that the median intervals between blood sampling and the delivery of the placenta were 29.0 days (IQR 21.5 to 35.0) and 28.5 days (IQR 23.0 to 34.0) in pathological and healthy pregnancies, respectively. We did not see a consistently changing pattern in the correlations between placental sFLT1 or PlGF and the maternal sFLT1:PlGF ratio when the interval between the 36wkGA blood sampling and delivery increased (Figure S3). These results would require a higher number of samples to be confirmed.

## Discussion

We confirm previous studies that the maternal sFLT1:PlGF ratio is elevated in the antenatal period in pregnancies complicated by preeclampsia and FGR. The main novel finding of the current analysis is that drivers for the increase in the ratio appear to differ in the two conditions. In preeclampsia, increased placental expression of sFLT1 appears to be the main driver for an elevated maternal sFLT1:PlGF ratio, whereas in FGR elevation of the sFLT1:PlGF ratio appears to be caused by reduced placental expression of PlGF. Hence, while both preeclampsia and FGR are clearly related to the function of the placenta, the underlying mechanisms are likely to differ. This is consistent with previous studies which have demonstrated an important role for spermine, a polyamine, in controlling trophoblast metabolism and demonstrated opposite associations between maternal circulating levels of a spermine metabolite and the risk of preeclampsia and FGR.^26, 27^ As both preeclampsia and FGR are major determinants of the global burden of disease,^28, 29^ mechanistic studies are required to better understand the commonalities and differences in the pathways leading to these two placentally-related complications.

The conclusion in relation to preeclampsia is consistent with a previous study which measured total and free PlGF in the serum of women with preeclampsia.^23^ These authors observed that among women with preeclampsia there was no difference in total PlGF but there were reduced levels of free PlGF, indicating that reduced levels detected by ELISA reflected increased binding by sFLT1. A point of difference between the current study and the previous report was that we found lower levels of PlGF in the placenta from women with preeclampsia, whereas Lecarpentier et al did not. Consistent with the protein results, we also found a significant decrease in placental PlGF mRNA levels in preeclamptic placentas compared to controls (Figure S4), Moreover, the previous study measured PlGF mRNA using RT-PCR and only studied 10 cases, whereas we measured PlGF protein and studied 82 cases.

In FGR, placental PlGF drives the higher maternal sFLT1:PlGF ratio. This seems in contrast with our results showing that placental PlGF levels are reduced in FGR cases compared to controls, but with a p value above the conventional threshold of 0.05 for statistical significance. We interpret this as being consistent with low PlGF in the placenta in a proportion of cases of FGR but the sample to sample variation reduced our statistical power to detect an effect. This sample to sample variation may reflect the phenotypic heterogeneity in FGR cases and other factors impacting on the placental levels of PlGF.

Our results on the altered circulating levels of sFLT1, PlGF and of the sFLT1:PlGF ratio in pregnancies with preeclampsia and FGR are consistent with previous results. The decrease in maternal PlGF levels are evident starting from the beginning of the second trimester in pregnancies with preeclampsia, while higher sFLT1 levels are detected in the third trimester leading to an increased sFLT1:PlGF ratio.^30, 31^ These and more recent data^1^ prompted the recommendation for clinical PlGF testing to help ruling out preeclampsia in women presenting with clinically suspected disease. Recently, a randomised controlled trial demonstrated that knowing PlGF levels reduced the time for clinical confirmation of the disease in women with suspected preeclampsia and improved maternal outcome.^32^ FLT1 and PlGF concentrations at 35-37wkGA in the highest and lowest 5th centile, respectively, were associated with FGR^33^ and abnormal maternal levels of these factors and of the sFLT1:PlGF ratio have been measured at various gestational ages in pregnancies with small infants, defined using multiple definitions of reduced fetal growth.^5, 34^

The current study had a number of strengths. First, the analyses were performed on a relatively homogenous group of women. Second, we were able to combine collection of blood prior to the onset of disease and correlate measurements made in the placenta obtained within 30 minutes of birth. This requires large scale recruitment coupled with efficient pipelines for sample collection. Third, antenatal measurements were made within a narrow gestational time window, mitigating the risk of confounding effects of gestational age related changes in protein levels in both maternal blood and placenta. Fourth, we had performed serial ultrasound and we were able to differentiate FGR and constitutively small (i.e. small for gestational age, SGA) infants across the entire cohort. Finally, the study was non-interventional, hence there was minimal capacity for the clinical interventions based on the results of research measurements to mask (or create) associations. However, further studies could address a number of weaknesses in the present analysis. As we confined the study to first pregnancies it is not clear whether these associations also apply to parous women. In the present study we focused on births at term. However, the pathophysiology of preeclampsia and FGR are thought to differ for preterm and term disease, hence further studies could address preterm complications. Finally, the current population was largely white European and it will be important to determine whether similar associations are observed in other groups.

In conclusion, we confirm that both preeclampsia and FGR are associated with an elevated sFLT1:PlGF ratio in the placenta and in maternal serum. However, the driver for the elevated ratio differs between the two conditions: in preeclampsia it is explained by increased placental sFLT1 whereas in FGR it is explained by decreased placental PlGF.

## Supplementary Material

Graphical Abstract

Supplemental Material

## Figures and Tables

**Table 1:** Characteristics of the study cohorts analyzed in this study by outcome status.

	PE	Control for PE	FGR	Control for FGR
	(n=82)	(n=82)	(n=50)	(n=50)
**Maternal characteristics**				
**Age, years**	30 (26 to 33)	30 (26 to 33)	32 (28 to 34)	30 (28 to 33)
**Age stopped full-time**	21 (17 to 22)	21 (18 to 22)	21 (18 to 23)	22 (21 to 24)
**education, years**				
Missing	6 (7)	1 (1)	1 (2)	0 (0)
**Body mass index, kg/m^2^**	27 (23 to 31)	26 (24 to 30)	23 (21 to 26)	24 (23 to 27)
**Smoker**	3 (4)	1 (1)	0 (0)	0 (0)
**Any alcohol consumption**	4 (5)	5 (6)	2 (4)	1 (2)
**Deprivation, score**	9.30	9.13	7.46	10.15
	(5.98 to 15.99)	(4.44 to 12.89)	(4.49 to 11.75)	(6.91 to 15.18)
Missing	3 (4)	4 (5)	3 (6)	3 (6)
**Deprivation, rank**	24765	24960	27063	23620
	(17270 to 28827)	(20405 to 30459)	(21655 to 30419)	(18028 to 27742)
Missing	3 (4)	4 (5)	3 (6)	3 (6)
**Deprivation score quartile**				
1 (lowest)	16 (20)	23 (28)	17 (34)	8 (16)
2	22 (27)	15 (18)	9 (18)	11 (22)
3	20 (24)	22 (27)	13 (26)	15 (30)
4 (highest)	21 (26)	18 (22)	8 (16)	13 (26)
Missing	3 (4)	4 (5)	3 (6)	3 (6)
**Ethnicity**				
Non white	4 (5)	5 (6)	2 (4)	4 (8)
White	77 (94)	76 (93)	45 (90)	46 (92)
Missing	1 (1)	1 (1)	3 (6)	0 (0)
**Married**	53(65)	47 (57)	37 (74)	41 (82)
**Birth outcomes**				
**Birthweight, g**	3530	3550	2782	3537
	(3080 to 3810)	(3380 to 3760)	(2500 to 2990)	(3400 to 3750)
**Birthweight, centile (population-based)**	49 (25 to 73)	51 (41 to 65)	5 (2 to 8)	49 (32 to 63)
**Gestational age, weeks**	40.1 (38.7 to 40.9)	40.4 (39.4 to 40.9)	40.5 (39.3 to 41.4)	40.6 (39.6 to 41.6)
**Time from 36wkGA sample to delivery, weeks**	4.1 (2.6 to 4.7)	4.0 (3.1 to 4.7)	4.2 (3.2 to 5.3)	4.1 (3.3 to 5.1)
Missing	4 (5)	1 (1)	2 (4)	1 (2)
**Method of delivery**				
Vaginal	47 (57)	54 (66)	35 (70)	35 (70)
Intrapartum caesarean	26 (32)	19 (23)	9 (18)	9 (18)
Pre-labor caesarean	9 (11)	9 (11)	6 (12)	6 (12)
**Severe PE**	33 (40)	0 (0)	2 (4)	0 (0)
**Uterine Doppler PI, highest decile**	16(20)	4 (5)	18 (36)	6 (12)
**Missing**	2 (2)	1 (1)	0 (0)	0 (0)
**Umbilical Doppler PI, highest decile**	10(12)	4 (5)	23 (46)	2 (4)
Missing	1 (1)	0 (0)	0 (0)	0 (0)
**AC growth velocity, lowest decile**	8 (10)	0 (0)	20 (40)	0 (0)
Missing	3 (4)	0 (0)	0 (0)	0 (0)

Data are expressed as median with 25^th^ and 75^th^ percentile or n with %, as appropriate. For fields where there is no category labelled ‘missing’, data are 100% complete. Maternal age was defined as age at recruitment. All other maternal characteristics were defined by self-report at the 20 weeks questionnaire, from examination of the clinical case record, or linkage to the hospital’s electronic databases. Socio-economic status was quantified using the Index of Multiple Deprivation (IMD) 2007, which is based on census data from the area of the mother’s postcode.^35^ Deprivation score is the combined sum of the weighted, exponentially transformed domain rank of the domain score, and higher values indicate more deprivation (also categorised into internal quartiles within the POP study cohort, 1=least deprived, 4=most deprived). Conversely, the most deprived area has the lowest rank and the least deprived area has the highest rank. Birth weight percentiles and z scores were calculated using a population-based UK reference.^21^ Severe PE is defined on the basis of the 2013 ACOG criteria.^19^ Uterine and umbilical Doppler PI and AC growth velocity including the highest and lowest deciles have been previously defined.^18^ PE: preeclampsia; FGR: fetal growth restriction; FTE: full time education; AC: abdominal circumference; PI: pulsatility index.F

## Pathophysiological Novelty and Relevance

### What is new?

- In healthy pregnancies, term placental levels of sFLT1 or PlGF did not correlate with maternal sFLT1, PlGF or the sFLT1:PlGF ratio at ˜36 weeks of gestation.
- In pregnancies with preeclampsia, placental sFLT1 was a stronger predictor of maternal serum PlGF levels than placental PlGF and drove the increase in sFLT1:PlGF.
- In pregnancies with FGR, placental PlGF was a stronger predictor of maternal serum PlGF levels than placental sFLT1 and drove the increase in sFLT1:PlGF.

### What is Relevant?

- Preeclampsia and FGR are associated with an increased risk of maternal and perinatal morbidity and mortality. Preeclampsia affects 5% of pregnant women, reaching peaks of 12-15% in undeveloped countries, and is responsible for the death of around 75,000 women and 500,000 babies worldwide each year. Unrecognized poor fetal growth accounts for approximately 30% of antepartum stillbirths.
- Our results identified different underlying mechanisms contributing to the complex pathophysiology of these placentally-related disorders of pregnancies.
- Unraveling the pathophysiology of preeclampsia and FGR can aid in designing appropriate interventions to improve maternal and fetal outcome in pregnancies with these conditions.

### Clinical/Pathophysiological Implications?

Although both preeclampsia and FGR are characterized by an elevated maternal sFLT1:PlGF ratio indicating a systemic angiogenic imbalance, our study demonstrates that the placenta differently contributes to the altered circulating angiogenic regulators observed in these conditions. These findings indicate that different strategies, aiming at reducing sFLT1 and increasing PlGF in the maternal circulation, should be pursued to design clinical interventions for preeclampsia and FGR, respectively.

## Perspectives

Our study demonstrates a different contribution of placental sFLT1 and PlGF towards the elevated sFLT1:PlGF ratio observed in pregnancies with preeclampsia and FGR. Increased placental sFLT1 drives the ratio in preeclampsia whereas decreased placental PlGF drives it in FGR. This study supports the view that, although preeclampsia and FGR share several clinical features, the underlying mechanisms leading to these two conditions are often not the same. Therefore, interventions aimed at reducing the elevated circulating levels of sFLT1 derived from the placenta could help reduce the severity of disease and prolong pregnancy in preeclamptic mothers. In pregnancies complicated with FGR, efforts should rather focus on increasing the maternal levels of PlGF.
