# Supplementary figures and images for: Increased placental soluble fms-like tyrosine kinase receptor-1 (sFLT1) drives the antiangiogenic profile of maternal serum preceding preeclampsia but not fetal growth restriction
Source: Hypertension. Author manuscript; Available in PMC 2023 Feb 1. (PMC9847691; doi:10.1161/HYPERTENSIONAHA.122.19482)

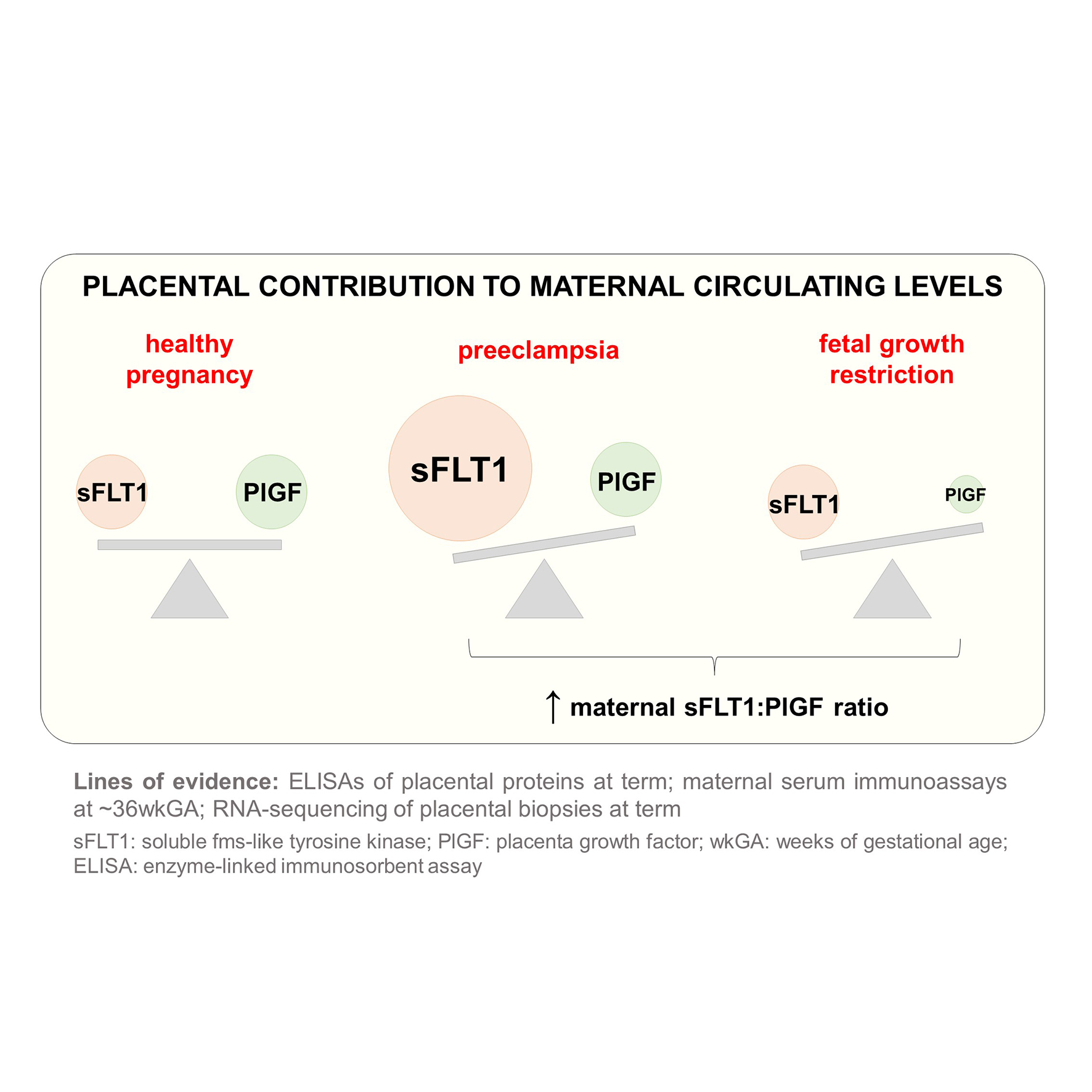

Supplement: Graphical Abstract [file EMS149699-supplement-Graphical_Abstract.jpg]
