# Supplementary material for: Increased placental soluble fms-like tyrosine kinase receptor-1 (sFLT1) drives the antiangiogenic profile of maternal serum preceding preeclampsia but not fetal growth restriction
Source: Hypertension. Author manuscript; Available in PMC 2023 Feb 1. (PMC9847691; doi:10.1161/HYPERTENSIONAHA.122.19482)
Supplement: Supplemental Material [file EMS149699-supplement-Supplemental_Material.docx]

Supplemental Material for:

**Increased placental soluble fms-like tyrosine kinase receptor-1 (sFLT1) drives the antiangiogenic profile of maternal serum preceding preeclampsia but not fetal growth restriction.**

Francesca Gaccioli^1,2^, PhD, Ulla Sovio^1,2^, PhD, Sungsam Gong^1^, PhD, Emma Cook^1^, MSc, D Stephen Charnock-Jones^1,2^, PhD, Gordon CS Smith^1,2^, MD PhD

^1^Department Obstetrics and Gynaecology, University of Cambridge, Cambridge, UK

^2^Centre for Trophoblast Research, University of Cambridge, Cambridge, UK

Correspondence to: Prof Gordon CS Smith, Department Obstetrics and Gynaecology, The Rosie Hospital, Robinson Way, Cambridge CB2 0SW, United Kingdom. Email address: [gcss2@cam.ac.uk](mailto:gcss2@cam.ac.uk); phone: +44 (0)1223 336871

**Table of Contents**

[Supplemental Methods 3](#_Toc107843994)

[*Total RNA-Seq analysis* 3](#_Toc107843995)

[*Quantification of the FLT1 transcript abundance levels* 3](#_Toc107843996)

[Supplemental Results 5](#_Toc107843997)

[*Analysis of FLT1 mRNA variants* 5](#_Toc107843998)

[*Effect of prolonged labor on placental sFLT1 and PlGF* 5](#_Toc107843999)

[References 7](#_Toc107844000)

[Supplemental Figures 8](#_Toc107844001)

# Supplemental Methods

## *Total RNA-Seq analysis*

We have previously peformed RNA-Seq analysis of 169 of the placental samples included in this study (n=79 from preeclamptic patients, n=3 from pregnancies with FGR, n=3 from pregnancies with both preeclampsia and FGR, and n=84 from controls). RNA extraction from placental samples and library preparation was performed as previously described.^1^ Briefly, placental biopsies were collected within 30 minutes of birth and flash frozen in RNAlater (ThermoFisher). Total placental RNA was extracted using the “mirVana miRNA Isolation Kit” (Ambion) followed by DNase treatment (“DNA-free DNA Removal Kit”, Ambion). Total RNA-libraries were prepared with the kit “TruSeq Stranded Total RNA Library Prep Kit with Ribo-Zero Human/Mouse/Rat” (Illumina) and sequenced (single-end, 125 bp) using a Single End V4 cluster kit and Illumina HiSeq2500 and HiSeq4000 instruments. Quality control and processing of the total RNA-Seq data has been previously described in detail.^1^ Placental mRNA levels are expressed as TPM (Transcript Per Million).

## *Quantification of the FLT1 transcript abundance levels*

We have used our entire RNA-Seq dataset (n=324) to estimate the proportion of FLT1 transcript variants in the placenta.^1^ The abundance of placental FLT1 mRNA variants was measured using Salmon^2^ with Ensembl annotation version 82. There were 8 Ensembl transcripts for FLT1 (ENSG00000102755), of which the following three were annotated as encoding soluble form of FLT1 proteins: ENST00000615840 (also known as sFlt1-i13 or sFlt_v1), ENST00000541932 (known as sFlt1-14 or sFlt1-15a or sFlt1_v2), and ENST00000539099. The abundance (measured in Transcript Per Million or TPM) of the aforementioned three transcripts were summed and used as the total abundance of FLT1 mRNA encoding sFLT1.

# Supplemental Results

## *Analysis of FLT1 mRNA variants*

We analyzed the FLT1 reads in our RNA-Seq dataset recently published (n=324).^1^ Among the 8 Ensembl FLT1 transcripts (see Supplemental Methods), the 3 most abundant transcripts were those encoding the soluble form of FLT1: ENST00000541932 (sFlt1-14; sFlt1-15a; sFlt1_v2; TPM=14.2), ENST00000615840 (sFlt1-i13; sFlt_v1; TPM=7.7), and ENST00000539099 (TPM=3.6). The abundance of the canonical transcript, encoding the membrane-bound form of FLT1 (ENST00000282397), was only 1.9 TPM. The abundance of the remaining transcripts encoding for the non-soluble forms was very marginal: ENST00000540678 (TPM=0.41), ENST00000543394 (TPM=0.11), ENST00000615611 (TPM=0.36), and ENST00000617835 (0.25). Overall, the abundance of the FLT1 transcripts encoding the soluble form was 8.3 times higher than those encoding the non-soluble forms, accounting for 89.2% of the total FLT1 mRNA.

## *Effect of prolonged labor on placental sFLT1 and PlGF*

The proportions of patients with vaginal delivery, intrapartum caesarean and pre-labor caesarean is 65%, 23% and 12%, respectively (see Table 1). For 186 deliveries the information on the duration of labor was available (5 women were part of both cohorts) and, of these, 104 women (56%) were in labor for more than 6h. We compared placental PlGF and sFLT1 protein expression in patients with >6h labor with those with shorter labor. This showed lower placental PlGF levels with prolonged labor (Figure S1). As there were similar proportions of cases and controls having labor shorter or longer than 6 hours, we do not think this changes our results: number (%) of patients in the PE cohort: 26 (45) controls and 17 (31) cases had labor <6h, while 32 (55) controls and 38 (69) cases had labor >6h; patients in the FGR cohort: 17 (47) controls and 21 (58) cases had labor <6h, while 19 (53) controls and 15 (42) cases had labor >6h.

# References

1. Gong S, Gaccioli F, Dopierala J, Sovio U, Cook E, Volders PJ, et al. The rna landscape of the human placenta in health and disease. *Nat Commun*. 2021;12:2639

2. Patro R, Duggal G, Love MI, Irizarry RA, Kingsford C. Salmon provides fast and bias-aware quantification of transcript expression. *Nat Methods*. 2017;14:417-419

# Supplemental Figures

**Supplemental Figure 1**

**Figure S1. Effect of prolonged labor on placental sFLT1 and PlGF.** Placental sFLT1 (A) and PlGF (B) protein levels at term are plotted based on the duration of labor (<6 hours *vs* >6 hours). A) n=78 when labor is <6 hours; n=99 when labor is >6 hours. B) n=81 when labor is <6 hours; n=100 when labor is >6 hours. Samples were removed from the analyses if measurements were not available or below the detection limit of the assay. Placental protein concentrations were log transformed and expressed as Z scores. P values obtained by unpaired t-test are provided. sFLT1: soluble fms-like tyrosine kinase 1; PlGF: placental growth.

**Supplemental Figure 2**

**Figure S2. Relationship between sFLT1 or PlGF mRNA levels and their corresponding proteins in the placenta and maternal circulation.** Placental mRNA levels encoding the soluble of FLT1 (A, n=166; B, n=153) and PlGF (C, n=169; D, n=153) are plotted against their protein levels in term placental and maternal serum samples at ~36 weeks of gestation. Samples were removed from the analyses if measurements were not available or below the detection limit of the assay. Maternal proteins are expressed as the multiple of the median (MoM) of control samples (adjusted for gestational age, maternal weight and storage time at measurement). Then MoM values and placental protein concentrations were log transformed and expressed as Z scores. Placental mRNA levels (measured in TPM, Transcript Per Million) were log transformed. Best fitting regression line (solid) and 95% confidence bands (dotted) are indicated. Text boxes report Pearson’s correlation coefficients (r) and P values. sFLT1: soluble fms-like tyrosine kinase 1; PlGF: placental growth.

**Supplemental Figure 3**

**Figure S3. Effect of an increasing interval between blood sampling and delivery on the relationship between placental sFLT1 and PlGF and the maternal sFLT1:PlGF ratio in pathological pregnancies.** Correlation analysis of the maternal sFLT1:PlGF ratio at ~36wkGA in relation to term placental levels of sFLT1 and PlGF in pregnancies with preeclampsia (A-B, circle symbols) and FGR (C-D, square symbols). Pearson’s correlation coefficients (r) and 95% confidence intervals (CI) are plotted. Samples were removed from the analyses if measurements were not available or below the detection limit of the assay. Maternal proteins are expressed as the multiple of the median (MoM) of control samples (adjusted for gestational age, maternal weight and storage time at measurement). Then both MoM values and placental protein concentrations were log transformed and expressed as Z scores. sFlt-1: soluble fms-like tyrosine kinase 1; PlGF: placental growth factor; CI: confidence intervals; FGR: fetal growth restriction.

**Supplemental Figure 4**

**Figure S4. Placental mRNA levels encoding PlGF in healthy and preeclamptic pregnancies.** mRNA levels, expressed as the log-transformed TPM (Transcript Per Million), were measured in term placentas from healthy and paired preeclamptic pregnancies (n=169). Boxes indicate the median, 25th and 75th percentiles. Whiskers extend to the minimum and maximum values. P values, obtained using paired 2-tailed t-test, are reported. PlGF: placental growth factor; CON: control/healthy pregnancy; PE: preeclampsia.
